# The role of radiotherapy in the updated German S2k guideline for management of Merkel cell carcinoma

**DOI:** 10.1007/s00066-023-02068-8

**Published:** 2023-03-28

**Authors:** Dirk Vordermark, Ulrike Höller

**Affiliations:** 1 Dept. of Radiation Oncology, Martin Luther University Halle-Wittenberg, Halle (Saale), Germany; 2 German Society of Radiation Oncology DEGRO, Berlin, Germany; 3 Universitätsklinik für Strahlentherapie, Universitätsklinikum Halle/Saale, Ernst-Grube-Str. 40, 06120 Halle/Saale, Germany

**Keywords:** Sentinel lymph node, Adjuvant therapy, Postoperative therapy, Tumor bed, Safety margin

## Abstract

Merkel cell carcinoma (MCC) is a radiosensitive tumor and the role of radiotherapy in the management of this disease was newly defined in the recently published update of the S2k guideline on Merkel cell carcinoma of the Association of Scientific Medical Societies in Germany (AWMF). While adjuvant radiotherapy of the tumor bed is broadly recommended, irradiation of the regional nodal region can be performed in patients with negative sentinel lymph nodes and high-risk factors. In patients with positive sentinel lymph nodes, it is an alternative to completion lymphadenectomy. The standard dose for adjuvant radiotherapy remains 50 Gy.

Merkel cell carcinoma (MCC) is a radiosensitive tumor and radiotherapy is a major modality in the management of MCC. Recommendations for radiotherapy in the newly updated German S2k guideline published by the Association of Scientific Medical Societies in Germany (*Arbeitsgemeinschaft Wissenschaftlicher Fachgesellschaften*, AWMF) [1] were revised based on recent findings from the literature since the previous version of this S2k guideline from 2018 [2]. We will discuss and explain the evidence on which the new recommendations are based. For each recommendation, the strength of recommendation is denoted with ↑↑ strong, ↑ moderate, or ↔ weak. The strength of agreement in the guideline group is denoted with ↑↑ strong (> 90% votes), ↑ moderate (> 75–90% votes), ↔ majority (> 50–75% votes) [1].

## Role of radiotherapy in the management of the primary tumor region

*Even after complete resection* *, adjuvant radiotherapy should be performed. Strength of recommendation: ↑, agreement: ↑↑.*

*Adjuvant radiotherapy of the tumor bed even after complete resection shall be performed with a total dose of 50* *Gy in single fractions of 2* *Gy including a safety margin of 3* *cm, which sufficiently treats the level of the skin (bolus). Strength of recommendation: ↑↑, agreement: ↑↑.*

Postoperative radiotherapy of the tumor bed following complete resection of MCC is an established standard of care. The updated S2k guideline recommends adjuvant radiotherapy of the tumor bed for all patients. Large datasets from tumor registries document a beneficial effect of adjuvant radiotherapy for patients in stage I (T1N0, tumor < 2 cm) and stage II (T2–4, N0) on overall survival [3, 4].

Omission of adjuvant radiotherapy in selected low-risk patients is currently not recommended in the updated German S2k guideline, but is discussed in the literature. For instance, a combination of all of the following factors is mentioned as “low risk” in the current Danish guidelines [5]: tumor ≤ 1 cm, negative margin, no lymphovascular invasion, negative sentinel lymph node biopsy, and no chronic immunosuppression. However, in Germany, adjuvant radiotherapy of the tumor bed is currently recommended for all patients. Specifically for MCC of the head and neck region, a matched-pair analysis of small tumors < 1 cm documented a 26% recurrence risk without radiotherapy vs. 0% with radiotherapy [6].

The surgical recommendations of the updated S2k guideline do not define a required extent of the surgical margin, but mention in the background text a surgical safety margin of at least 1 cm in patients with stage I tumors (maximum diameter 2 cm) and of at least 2 cm in patients with stage II tumors (diameter > 2 cm). The safety margin for adjuvant radiotherapy of the tumor bed is not uniformly defined in international recommendations and ranges from 1–2 cm in the Danish guidelines [5] to 5 cm in the National Comprehensive Cancer Network (NCCN) guidelines [7]. Its purpose is not primarily to address uncertain resection margins of the primary tumor but rather to eliminate microscopic satellite metastases. For the purpose of the current S2k guideline, an intermediate safety margin of 3 cm around the primary tumor region and scar was recommended. For all radiotherapy techniques, an adequate surface dose has to be guaranteed and this is usually achieved by use of a bolus.

The standard dose for adjuvant radiotherapy of the tumor bed continues to be 50 Gy in daily fractions of 2 Gy. Although no new high-level evidence was found to support this dose level, 50 Gy was the standard dose in several retrospective analyses, including a series of low-risk head and neck MCC with 0% local recurrence (compared to 26% in the reference group treated with surgery alone) [6] and our own experience with a disease-specific survival of 95.7% in a mixed-stage cohort treated with adjuvant radiotherapy [8].

## Role of radiotherapy in the management of the lymph node region

*In case of a negative sentinel lymph node and increased risk of a false negative finding or of a recurrence (maximum tumor diameter >* *2* *cm), radiotherapy of the involved lymph node region can be performed. Strength of recommendation: ↑, agreement: ↑↑.*

*In case of a positive sentinel lymph node biopsy (micrometastases), a therapeutic lymph node dissection or radiotherapy of the involved region should be performed. Strength of recommendation: ↑, agreement: ↑.*

*In the presence of clinically manifest lymph node metastases and an M0 situation, a therapeutic lymph node dissection or radiotherapy alone of the involved lymph node region should be considered. Strength of recommendation: ↑, agreement: ↑. In situations with an increased risk of recurrence (e.g., extracapsular extension), the combination can be applied. Strength of recommendation: ↔, agreement: ↑↑.*

*In case of subclinical tumor, radiotherapy should be performed with a total dose of 50–56* *Gy, in case of macroscopic/clinically detectable tumor, with ≥* *56* *Gy. Strength of recommendation: ↑, agreement: ↑↑.*

The surgical part of the new S2k guideline recommends a sentinel lymph node biopsy in cases where clinical examination and imaging document an N0M0 situation. The further management after sentinel lymph node biopsy is not clear. Adjuvant radiotherapy including not only the tumor bed but also routinely the draining lymph node region has been a standard of care since the randomized trial of Jouary et al., who showed a reduction of regional recurrences from 16.7% (with radiotherapy of the tumor bed only) to 0% (with radiotherapy of the tumor bed and the lymph node region) in stage I patients (tumor < 2 cm, N0) [9]. However, this trial was completed before introduction of sentinel lymph node biopsy in MCC.

General omission of adjuvant regional lymph node irradiation (i.e., radiotherapy of the tumor bed alone) in the situation of histopathologically negative sentinel lymph nodes has been questioned by our own experience showing a 5-year risk of regional relapse in such patients of 33% with radiotherapy and of 0% with radiotherapy [10]. Other retrospective analyses also documented a prognostic benefit from radiotherapy of the regional lymph nodes even in the setting of negative sentinel lymph nodes [11], leading to the recommendation in the S2k guideline that adjuvant radiotherapy of the lymph node region in the presence of negative sentinel lymph nodes *can *be performed in high-risk situations (defined in the recommendations as primary tumors of > 2 cm; in the background text the uncertain lymphatic drainage in the head and neck region is also mentioned as a high-risk factor).

In the case of a positive sentinel lymph node, lymphadenectomy does not necessarily have to be performed, but radiotherapy is alternatively recommended in the updated S2k guideline. According to new data from retrospective studies and prospective databases, the outcomes after radiotherapy are not inferior to those of lymphadenectomy, similar to the situation in other tumor entities [12, 13]. For instance, in a dataset where completion lymph node dissection (CLND) was recommended in patients with acceptable perioperative risk and therapeutic radiotherapy, in those with high perioperative risk, the 3‑year nodal recurrence-free survival was 76% after CLND and 91% after radiotherapy (*p* = 0.3) [14]. If patients have additional positive lymph nodes in the specimen of the CLND, especially with extracapsular extension, additional regional radiotherapy after lymphadenectomy can be applied [15].

## Role of radiotherapy in the management of macroscopic tumor or of distant metastases

*For distant metastatic MCC, radiation therapy is employed as part of a multimodal treatment approach that also includes surgical excision and/or systemic chemo- or immunotherapy. The decision on whether to employ this modality has to be made on a case-by-case basis, and the procedure is usually performed with palliative intent. Agreement: ↑↑.*

The recommended dose level for macroscopic tumor is still ≥ 56 Gy (see section “Role of radiotherapy in the management of the lymph node region”). The efficacy of radiotherapy alone in inoperable MCC was analyzed in a recent systematic review [18]: in patients with stages I and II (i.e., node-negative tumors), median doses of 55 Gy (local radiotherapy only) and 65 Gy (local and regional radiotherapy) resulted in relapse rates of 25 and 21%, respectively, indicating the curative potential of radiation monotherapy without surgery. In stage III (node-positive) patients, 40% remained relapse free after monotherapy alone.

For the setting of metastatic disease, the S2k guideline recommends immunotherapy targeting PD-1/PD-L1 as superior to chemotherapy. While potential for a benefit from combinations of radiotherapy and immunotherapy in MCC is seen, the very limited clinical data did not justify a change of the previous recommendation that radiotherapy can be integrated into multimodal concepts on an individual basis [16, 17].
